# Loss of Predicted Cell Adhesion Molecule MPZL3 Promotes EMT in Ovarian Cancer

**DOI:** 10.1158/2767-9764.CRC-24-0591

**Published:** 2025-07-21

**Authors:** Ya-Yun Cheng, Beth L. Worley, Zaineb Javed, Amal T. Elhaw, Priscilla W. Tang, Sarah Al-Saad, Shriya Kamlapurkar, Sierra R. White, Apoorva Uboveja, Karthikeyan Mythreye, Katherine M. Aird, Traci A. Czyzyk, Nadine Hempel

**Affiliations:** 1 Division of Malignant Hematology & Medical Oncology, Department of Medicine, UPMC Hillman Cancer Center, University of Pittsburgh, Pittsburgh, Pennsylvania.; 2 Department of Pharmacology, College of Medicine, Pennsylvania State University, Hershey, Pennsylvania.; 3 Department of Pharmacology & Chemical Biology, UPMC Hillman Cancer Center, University of Pittsburgh School of Medicine, Pittsburgh, Pennsylvania.; 4 Department of Pathology and O’Neal Comprehensive Cancer Center, Heersink School of Medicine, University of Alabama at Birmingham, Birmingham, Alabama.; 5 Department of Anesthesiology & Perioperative Medicine, Penn State University College of Medicine, Hershey, Pennsylvania.

## Abstract

Myelin protein zero-like 3 (MPZL3) is an immunoglobulin-containing transmembrane protein with predicted cell adhesion molecule function. Loss of 11q23, in which the *MPZL3* gene resides, is frequently observed in cancer. Yet the role and consequences of altered MPZL3 expression have not been explored in tumor development and progression. We addressed this in ovarian cancer, in which both MPZL3 amplification and deletions are observed in respective subsets of high-grade serous specimens. Whereas high and low MPZL3-expressing populations are similarly observed in primary ovarian tumors from an independent patient cohort, metastatic omental tumors largely display decreased MPZL3 expression, suggesting that MPZL3 loss is associated with metastatic progression. MPZL3 knockdown leads to an increase in EMT gene expression in OVCAR4 and OVCA433 cell lines, a transcript signature that is associated with poor patient outcomes. MPZL3 promotes homotypic cancer cell adhesion, and decreasing MPZL3 expression enhances invasion and clearance of mesothelial cell monolayers. Conversely, MPZL3 loss abrogates cell-cycle progression and proliferation, with cells adopting senescence features. This was associated with decreased sensitivity to cisplatin and reduced DNA damage and apoptosis in response to treatment in OVCAR4 cells. Our study suggests that decreased expression of the predicted adhesion molecule MPZL3 is associated with low proliferation but increased metastatic potential during ovarian cancer tumor progression.

**Significance:**

This work presents novel findings that decreased expression of the potential cell adhesion molecule MPZL3 is a phenotype of ovarian cancer progression and metastasis.

## Introduction

Ovarian cancer is the second most lethal gynecologic malignancy in the United States, responsible for more than 12,000 deaths annually. Patients with stage IV high-grade serous adenocarcinoma, which is the most common ovarian cancer histologic subtype, are faced with a 5-year overall survival rate of less than 27% (1, 2). More than 60% of patients are diagnosed after stage III, due in part to both lack of effective screening methods and asymptomatic disease progression until metastasis to the peritoneal cavity (1). Ovarian cancer frequently undergoes metastasis through transcoelomic pathways. This process involves the detachment of cells from the primary tumor site, leading to their spread as either individual cells or spheroids into the peritoneal cavity, a process that is significantly influenced by alterations in cell–cell and cell–extracellular matrix adhesion (3–7). Depending on the type of cell adhesion molecule (CAM), both loss and gain of CAMs can contribute to metastatic progression.

Myelin protein zero-like 3 (MPZL3) is an immunoglobulin (Ig) domain–containing transmembrane protein that shares sequence homology with junctional adhesion molecule family members, including V-set and Ig domain–containing 1 (VSIG1). The function of MPZL3 has primarily been investigated through studies in murine knockout models, in which it has been associated with epidermal barrier formation, sebaceous gland differentiation, hair follicle cycling, and metabolic regulation (8–15). In addition, we have shown that even transient knockdown of MPZL3 can prevent the negative metabolic effects of a high-fat diet in mice (8). Furthermore, MPZL3 can also localize to mitochondria (16). However, it remains largely unclear whether and how these functions relate to MPZL3’s role as a potential CAM.

Junctional adhesion molecules that share sequence homology with MPZL3 and other MPZ myelin zero-like proteins are often highly expressed in normal polarized epithelial and endothelial cells. The loss of VSIG1 was shown to be a feature of gastric, lung, and esophageal cancer cells and necessary for metastasis (17). Whether MPZL3 has similar properties in tumor cells has not been explored (10). Interestingly, the *MPZL3* locus (11q23.3) is susceptible to chromosomal loss in multiple cancer subtypes (18–21), and loss of heterozygosity of this location is a frequent event in ovarian cancer (19). This suggests that loss of *MPZL3* may be a phenotype of cancer. Previous research indicates varying MPZL3 expression patterns across different cancer types, suggesting that the role of MPZL3 is context-dependent (22). High MPZL3 expression has been associated with poor prognosis in breast cancer and shown to drive the proliferation of *MET*-amplified cancer cell lines (22, 23).

In this study, we set out to explore the role of MPZL3 in ovarian cancer and found that loss of MPZL3 results in loss of homotypic cell adhesion and a more invasive phenotype associated with epithelial to mesenchymal transition (EMT). In addition, MPZL3 knockdown slows cell-cycle progression, promotes a senescent-like phenotype, and may contribute to loss of chemosensitivity to DNA-damaging agents. These data demonstrate that decreased MPZL3 expression is a phenotype of ovarian cancer tumor progression and metastasis and may contribute to treatment failure in patients with advanced-stage disease.

## Materials and Methods

### Cell culture

All cell lines were cultured in a 5% CO_2_ environment at 37°C. OVCAR4 cells (MilliporeSigma, SCC258, RRID: CVCL_1627) were cultured in RPMI 1640 (Gibco, 11875) supplemented with 2 mmol/L glutamine (Gibco, 25030081), 10% FBS (Avantor Seradigm, 1500-500), and 0.25 U/mL insulin (Gibco, 12585014). OVCA433 (RRID: CVCL_0475) was kindly provided by Dr. Susan K. Murphy (Duke University) and cultured in RPMI 1640 medium (Corning, 10-040-CV) supplemented with 10% FBS. SKOV3-Luc with stably expressed Luciferase was a gift from Dr. Karthikeyan Mythreye (the University of Alabama at Birmingham) and maintained in RPMI 1640 medium (Corning, 10-040-CV) supplemented with 10% FBS. 293FT (CVCL_6911) was purchased from Invitrogen (R70007) and cultured in DMEM (Corning, 10-017-CV) supplemented with 10% FBS. NIH:OVCAR3 (OVCAR3; RRID: CVCL_0465) and OV90 (RRID: CVCL_3768) cells were purchased from the ATCC (HTB-161, CRL-3585). OVCAR3 was maintained in RPMI 1640 medium (Gibco, 11875) supplemented with 10% FBS and 0.01 mg/mL bovine insulin (MilliporeSigma, I0516). OV90 was maintained in a 1:1 mixture of MCDB 105 medium (Cell Applications, 117-500) with 1.5 g/L sodium bicarbonate (Gibco, 25080094) and Medium 199 (Corning, 10-060-CV) with 2.2 g/L sodium bicarbonate, supplemented with 15% FBS. FT282 (CVCL_A4AX) was kindly provided by Dr. Ronny Drapkin (University of Pennsylvania) and cultured in 50% DMEM and 50% Ham’s F-12 medium (Corning, 10-090-CV) supplemented with 2% FBS. GFP-labeled mesothelial cells, ZTGFP cells, were a gift from Dr. Ioannis Zervantonakis (University of Pittsburgh; ref. 24) and maintained in 1:1 mixture of Medium 199 (Corning, 10-060-CV) and MCDB105 (Cell Applications, 117-500) media supplemented with 10% FBS and 1% penicillin–streptomycin (Gibco). Cells are routinely tested for *Mycoplasma* using EZ-PCR Mycoplasma Detection Kit (Captivate Bio, 20-700-20) and authenticated using short tandem repeat sequencing (Labcorp). Cells were used within 15 passages following thawing.

### Cloning and lentiviral transduction

MPZL3 short hairpin RNAs (shRNA) were from the TRC library (MilliporeSigma) with the following targeting sequences: shMPZL3 #1: GAG​TCA​CCT​AAA​GAC​AGG​AAA (TRCN0000137304); shMPZL3 #2: GGG​ATG​CAT​CTA​TAA​GTA​TAA (TRCN0000413087); and shMPZL3 #3: GCA​GCC​ACA​CAG​TAT​CAA​TAT (TRCN0000137252). pLKO.1 scramble (scr) shRNA (RRID: Addgene_1864) and pLKO.1 control shRNA (RRID: Addgene_8453) were used as controls. The expression or shRNA plasmids were cotransfected with the virus package plasmids, psPAX2 (RRID: Addgene_12260) and pMD2.G (RRID: Addgene_12259), to 293FT cells for virus production. Cells were seeded on six-well plates the day before virus transduction. A measure of 200 μL of virus was added to the cells with polybrene (MilliporeSigma, TR-1003; 0.8 μg/mL) and incubated for 24 hours. Drug selection was performed 3 days after transduction, with the following concentrations: OVCA433, 2 μg/mL of puromycin (Gibco, A1113803); SKOV3-Luc, 3 μg/mL of puromycin; and OVCAR4, 2 μg/mL of puromycin. To reduce the risk of knockdown loss and minimize potential compensatory effects from long-term culture, all functional assays were performed shortly after puromycin selection.

### RNA isolation and semi-quantitative RT-PCR

Total RNA was isolated using Direct-zol RNA Miniprep Kit (Zymo Research, R2052) and used for cDNA synthesis (Quantabio: 95047) based on the manufacturer’s instruction. cDNA samples were analyzed by semi-quantitative RT-PCR using Bio-Rad CFX Opus 96 Real-Time PCR System with PowerUp SYBR Green Master Mix (Applied Biosystems) or iTaq Universal SYBR Green Supermix (Bio-Rad). The following primer sequences were used: MPZL3 sense: 5′- ATG​CCC​ATG​TCC​GAG​GTT​ATG -3′; MPZL3 antisense: 5′- GGA​GGG​CGA​TAT​GTC​CAG​TCT-3′; GAPDH sense: 5′-GAG​TCA​ACG​GAT​TTG​GTC​GT-3′; GAPDH antisense: 5′- TTG​ATT​TTG​GAG​GGA​TCT​CG -3′; ACTB sense: 5′- AGA​GCT​ACG​AGC​TGC​CTG​AC-3′; ACTB antisense: 5′- AGC​ACT​GTG​TTG​GCG​TAC​AG-3′; TBP sense: 5′- TTG​GGT​TTT​CCA​GCT​AAG​TTC​T-3′; and TBP antisense: 5′- CCA​GGA​AAT​AAC​TCT​GGC​TCA-3. RNA expression was normalized to the geometric mean of the reference genes (GAPDH, ACTB, and TBP), and relative changes between samples were calculated by normalizing to scr control cells using the 2^−ΔΔCT^ formula.

### Patient data analysis

*MPZL3* copy-number alteration (CNA) incidence was examined on cBioPortal (RRID: SCR_014555) with the following datasets: pan-cancer analysis of whole genomes [International Cancer Genome Consortium/The Cancer Genome Atlas (TCGA); ref. 25] and ovarian serous cystadenocarcinoma (TCGA, PanCancer Atlas). Overall survival and transcriptome data for the ovarian serous cystadenocarcinoma (TCGA, PanCancer Atlas) dataset were obtained from cBioportal (RRID: SCR_014555). For analyzing transcriptome changes according to the expression of MPZL3, the data were divided into quantiles based on the expression level of *MPZL3* RNA. By comparing the first (low MPZL3) and the fourth (high MPZL3) quantiles, genes that showed significantly higher expression in each group were selected for further analysis. Pathway analysis was performed using Molecular Signatures Database (MSigDB) analysis to compute overlaps between the selected genes and the gene sets in MSigDB (26–28). In this study, we applied hallmark gene set for analysis (28).

### Tumor specimens

Specimens of ovarian tumor and omental tumor from patients with high-grade serous ovarian cancer and their matched normal fallopian tube control samples were obtained through an honest broker from the ProMark biospecimen bank at the University of Pittsburgh, with Institutional Review Board approval from the University of Pittsburgh (protocol # STUDY21050194). RNA was isolated, and RT-PCR was carried out as above. Data were first normalized to housekeeping genes, as above, and MPZL3 expression in tumor samples (T-OV and T-OM) expressed relative to matched normal fallopian tube samples from the same patient using the 2^−ΔΔCT^ method.

### RNA sequencing

OVCAR4 cells were transduced with scramble (scr) or MPZL3 shRNA (sh #1 and sh #2) virus and selected with puromycin for 3 days. OVCA433 cells were transduced to express empty vector control or MPZL3 shRNA (sh #1 and sh #3). Three biological replicates were performed for each condition. To minimize off-target effects and assess knockdown robustness across different cell lines, distinct shRNAs were used for OVCAR4 and OVCA433 cells, with one shRNA sequence shared between both lines. This strategy enabled evaluation of three independent shRNAs while allowing assessment of potential cell line–specific responses. Total RNA was extracted from cells using Direct-zol RNA Miniprep Kit (Zymo Research, R2052). The samples were sequenced using the Illumina NovaSeq 6000 platform (RRID: SCR_016387), and sequence alignment was conducted with HISAT2 mapping tool (RRID: SCR_015530). For each cell line, two MPZL3 shRNA-treated samples were grouped as the knockdown condition and compared with the corresponding scr control group. Differential expression analysis was performed between the knockdown and control groups using the DESeq2 R package (RRID: SCR_015687). The adjusted *P* value ≤0.05 was used to define differentially expressed genes.

### Protein isolation and Western blotting

Cells were scraped and harvested in RIPA buffer (Thermo Fisher Scientific, 89901) with protease and phosphatase inhibitors (Thermo Fisher Scientific, 78443). Protein concentration was measured with the Bradford protein assay (Bio-Rad, 5000006). Thirty μg of total protein were loaded to 4% to 20% SDS-PAGE gels (Bio-Rad) and transferred to polyvinylidene difluoride membranes (Thermo Fisher Scientific, PI88520) after electrophoresis. The membranes were blocked with 5% nonfat milk (Bio-Rad, 1706404) in TBS containing 0.1% Tween-20 (MilliporeSigma, 900-64-5) for 30 minutes and incubated with primary antibodies at 4°C overnight. The primary antibodies used in this study included MPZL3 (Novus Biologicals, NBP2-84169, RRID: AB_3425834, 1:500), N-cadherin (Cell Signaling Technology, #14215, RRID: AB_2798427, 1:1,000), vimentin (VIM; Cell Signaling Technology, #5741, RRID: AB_10695459, 1:1,000), GAPDH (Santa Cruz, #sc-47724, RRID: AB_627678, 1:1,000), phospho-histone H2A.X (Ser139; MilliporeSigma, 05-636, RRID: AB_309864, 1:1,000), histone H2A.X (Santa Cruz, #sc-517336, RRID: AB_3675923, 1:1,000), and vinculin (MilliporeSigma, V9131, RRID: AB_477629, 1:1,000). The next day, after washing with 0.1% TBS–Tween-20, the membranes were incubated with horseradish peroxidase–conjugated secondary antibodies (Cytiva, NA931, RRID: AB_772210 and NA934, RRID: AB_772206, 1:10,000) at room temperature for 1 hour. After washing, the signals were detected using SuperSignal West Femto Maximum Sensitivity Substrate (Thermo Fisher Scientific, 34096) and ChemiDoc XRS+ system (Bio-Rad, RRID: SCR_019690).

### Adhesion assay

Parental OVCAR4 or mesothelial ZTGFP cells were seeded in 96-well plates. The following day, OVCAR4 cells expressing scr control or MPZL3 shRNA (sh #1 and sh #2) were stained with CellTrace Far Red (Invitrogen, C34572) and added as a suspension to the preseeded 96-well plates. After incubating for 30 minutes at 37°C, nonadherent cells were washed off with PBS 3 times, each for 5 minutes. The adherent cells were then examined under a microscope (Leica Thunder Imager) and analyzed using Fiji software (RRID: SCR_002285). The number of adherent cells was then normalized to the corresponding seeding control, in which cells were plated but not subjected to the washing step, to account for variations in initial cell seeding and calculate relative adhesion efficiency.

### Spheroid formation and mesothelial clearance assay

OVCAR4 cells expressing scr control or MPZL3 shRNAs (sh #1 and sh #2) were stained with CellTrace Far Red (Invitrogen, C34572), seeded into 96-well ultra low attachment plates (Corning, 7007), and incubated overnight to form spheroids. The following day, the spheroids were transferred to a preseeded 96-well plate containing a layer of ZTGFP cells. After 48 hours of incubation, images were captured using Leica Thunder Imager and analyzed with Fiji software (RRID: SCR_002285) to assess the areas cleared by the spheroids.

### Growth assay and IC_50_ value analysis

Cells were seeded into 96-well plates in 100 μL medium with the following conditions: OVCA433, 500 cells/well; OVCAR4, 1,000 cells/well; and SKOV3-Luc, 500 cells/well. Cell growth assay was performed using FluoReporter Blue Fluorometric dsDNA Quantitation Kit (Invitrogen, F2962). One 96-well plate was harvested, with medium removed, and stored at −80°C in a freezer every 24 hours. A total of five plates were collected and proceeded to Hoechst 33258 staining according to the manufacturer’s instruction. The DNA quantification was measured using a fluorescence plate reader (PerkinElmer) with excitation at 360 nm and detection at 460 nm. The cell growth rate was calculated by normalizing the values to day 1. IC_50_ value analysis was performed using OVCAR4 cells with or without MPZL3 knockdown. The cells were seeded and treated with paclitaxel (MilliporeSigma), cisplatin (MilliporeSigma), or olaparib (Selleck Chemicals, S1060) the next day and then incubated for 72 hours. The plates were harvested and measured using FluoReporter Blue Fluorometric dsDNA Quantitation Kit. IC_50_ values were calculated based on the percentage survival relative to untreated cells.

### Cell-cycle analysis

OVCAR4 cells were seeded in six-well plates at a density of 125,000 cells per well and incubated overnight. The following day, the cells were subjected to a double thymidine block for G1/S phase synchronization. Briefly, cells were treated with 2 mmol/L thymidine (Sigma-Aldrich, T9250) for 18 hours, released for 8 hours, and then incubated again with 2 mmol/L thymidine for an additional 16 hours. Afterward, the cells were placed in fresh media and harvested at 0, 3, 6, 9, and 24 hours. Following staining with propidium iodide (Abcam, ab139418), cell-cycle status was assessed by flow cytometry (Beckman Coulter, CytoFLEX, RRID: SCR_019627) and analyzed using FlowJo software (BD Life Sciences, RRID: SCR_008520).

### Caspase 3/7 assay

OVCAR4 cells were seeded in 96-well plates at a density of 2,000 cells per well. The following day, cells were treated with 1.3 μmol/L cisplatin [cis-diammineplatinum(II) dichloride; Sigma] or 3.9 μmol/L olaparib (Selleck Chemicals, S1060) for 72 hours. Caspase 3/7 activity was then assessed using the Caspase-Glo 3/7 Assay (Promega, G8091) according to the manufacturer’s protocol. Cells were incubated with an equal volume of lysis reagent for 1 hour at room temperature, protected from light. The samples were transferred to white 96-well plates (BrandTech, BRA-781605), and bioluminescence signals were measured using a GloMax Explorer plate reader (Promega).

### Senescence-associated β-galactosidase activity assay

OVCAR4 or OVCA433 cells were seeded in 12-well plates at a density of 75,000 or 50,000 cells per well, respectively, and incubated for 48 hours. The cells were then fixed with a solution containing 0.2% glutaraldehyde (Polysciences, 01909-100) and 2% formaldehyde (VWR, 0493-500) for 5 minutes. Afterward, a β-galactosidase staining solution containing 150 mmol/L NaCl (MilliporeSigma), 2 mmol/L MgCl_2_ (MilliporeSigma), 5 mmol/L K_3_Fe(CN)_6_ (MilliporeSigma), 5 mmol/L K_4_Fe(CN)_6_ (MilliporeSigma), 40 mmol/L Na_2_HPO_4_ (MilliporeSigma; pH 5.4 for OVCAR4 and pH 5 for OVCA433), and 20 mg/mL X-gal (MilliporeSigma, B4252) was added to the cells. The cells were incubated in a non-CO_2_ incubator at 37°C for 16 hours, then washed three times with ddH_2_O, and then stored in 50% glycerol. Images were taken using a microscope (Invitrogen, EVOS XL Core) at 10× magnification and analyzed with Fiji software (RRID: SCR_002285).

### Immunofluorescence

OVCAR4 cells were seeded on eight-well chambered cell culture slides (Falcon, 08-774-26) for overnight incubation. The next day, the cells were treated with 5 μmol/L cisplatin [cis-diammineplatinum(II) dichloride; Sigma] or 3.9 μmol/L olaparib (Selleck Chemicals, S1060) for 72 hours. The cells were fixed with 4% paraformaldehyde (BeanTown Chemical, 30525-89-4) for 10 minutes, permeabilized with 0.25% Triton X-100 (Thermo Fisher Scientific, BP151500) in PBS (Corning, 21-040-CV) for 10 minutes, and incubated with blocking buffer containing 2% goat serum/PBS (Cell Signaling Technology, 5425) for 30 minutes at room temperature. Then, the cells were incubated with anti–phospho-histone H2A.X (Ser139) antibody (MilliporeSigma, 05-636, RRID: AB_309864, 1:350) in blocking buffer at 4°C overnight. The next day, after washing with PBS three times, the blocking buffer with goat anti-rabbit Alexa Fluor 488–conjugated (Thermo Fisher Scientific, A-11008, RRID: AB_143165, 1:1,000) antibody was added, incubating at room temperature for 1 hour protected from light. After washing, the cover slips were mounted with ProLong Gold Antifade Reagent with 4′,6′-diamidino-2-phenylindole(Cell Signaling Technology, 8961) and dried at room temperature overnight protected from light. The signals were examined using Leica Thunder Imager at 20× magnification and analyzed by Fiji software (RRID: SCR_002285).

### Tumor xenografts

Approval for animal studies was sought from the University of Pittsburgh Institutional Animal Care and Use Committee prior to study commencement (approved protocol IS00023174). All animals were housed in barrier facilities for immunodeficient mice in the University of Pittsburgh Animal Faculty, and mouse husbandry and experiments were performed in accordance with the guidelines of the Laboratory Animal Ethics Committee of the University of Pittsburgh. OVCAR4 cells expressing scr or MPZL3 shRNA (sh #1) were mixed with Matrigel (Corning, 354248) for both right and left subcutaneous (flank) injections into female Nod/SCID gamma mice (The Jackson laboratory; scr: *n* = 10, sh #1: *n* = 10, 5 × 10^6^ cells per injection). Mice were randomized prior to drug treatment. Subcutaneous tumor growth was monitored using caliper measurements twice weekly, and tumor volumes were calculated according to the formula V = ½ [length (longer diameter) × width (shorter diameter)^2^]. When the average tumor volume in each group reached 50 mm^3^, mice were injected intraperitoneally with cisplatin or vehicle control (saline) twice per week. The cisplatin dosing regimen consisted of 2 mg/kg body weight for 5 doses, followed by 3 mg/kg body weight for 2 doses, a pause for 3 doses, and then 2 mg/kg body weight for the final 2 doses (Supplementary Fig. S5A). One mouse was removed from the study prior to drug treatment because of illness unrelated to tumor burden. One mouse died because of cisplatin toxicity early on and thus was excluded from the final analysis. The mice were euthanized after completing the treatment regimen (a total of 12 doses, including 3 pauses), and the xenografted tumors were resected and measured. To determine chemotherapy response, the tumor growth rates were calculated by dividing the final tumor volumes by the average tumor volumes from three measures: 3 days before treatment, on the day treatment began, and 4 days after treatment began.

### Statistical analysis

Unless otherwise noted, the data presented in this study are based on at least three independent experiments and are expressed as the mean ± SEM. All statistical analyses were performed using GraphPad Prism 10 (RRID: SCR_002798) and were selected based on the experimental design, as indicated. Statistical significance was defined as *P* < 0.05.

### Data availability

The RNA sequencing (RNA-seq) data generated in this study are publicly available in Gene Expression Omnibus (GSE296256 and GSE296257). Other data from this study are available from the corresponding author upon request.

## Results

### *MPZL3* gene displays CNAs in cancer and MPZL3 expression is decreased in omental metastatic lesions

**Figure 1:**
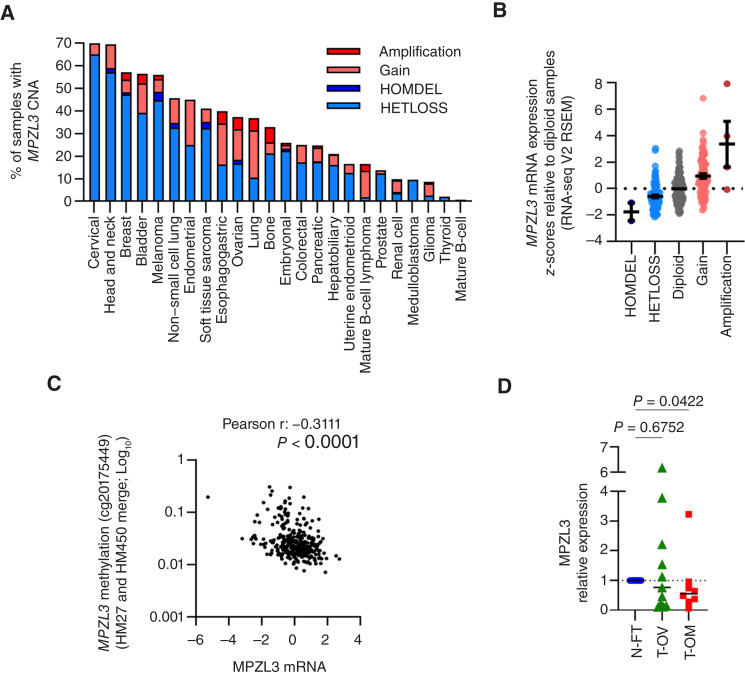
MPZL3 expression in ovarian cancer tissues. **A,** Percentage of tumor samples displaying *MPZL3* CNA based on International Cancer Genome Consortium/TCGA pan-cancer analysis of whole genomes. **B,** MPZL3 mRNA expression and *MPZL3* CNA in ovarian adenocarcinoma samples (TCGA, PanCancer Atlas). **C,** MPZL3 methylation is negatively associated with MPZL3 mRNA expression (TCGA, PanCancer Atlas). **D,** MPZL3 transcript levels detected by semi-quantitative RT-PCR in high-grade serous ovarian (T-OV, *n* = 11) and omental tumors (T-OM, *n* = 8) compared with normal fallopian tube tissues (N-FT, *n* = 14; Kruskal–Wallis multiple comparison test *P* = 0.07; Dunn multiple comparison test *P* values shown). HOMDEL, deep deletion; HETLOSS, shallow deletion.

The *MPZL3* gene is located on chromosome 11q23.3, a region known to be susceptible to chromosomal loss across multiple cancer subtypes (18–20). Loss of heterozygosity at 11q23 has been observed in breast, lung, colorectal, and cervical cancers (21, 29–34) and is a frequent event in ovarian carcinoma (19). CNA analysis of International Cancer Genome Consortium/TCGA PanCaner Atlas data (25) across 24 tumor types found that many tumor samples exhibit *MPZL3* copy-number loss (Fig. 1A). Interestingly, esophageal, lung, and ovarian tumors display both loss and gain of *MPZL3.* Approximately 30% of ovarian adenocarcinoma samples have *MPZL3* heterozygous loss, whereas 25% exhibit chromosomal gain (Supplementary Fig. S1A), and these CNAs correlate with MPZL3 mRNA transcript levels (Fig. 1B). In addition, MPZL3 transcript expression was negatively associated with gene methylation status (Fig. 1C), suggesting that CNAs and epigenetic mechanisms contribute to downregulation of MPZL3 in ovarian tumors. Analysis of patient survival demonstrated that *MPZL3* copy-number loss was associated with a 9.2 months decrease in overall patient survival, although this association was not statistically significant (*P* = 0.2621; Supplementary Fig. S1B). To further assess the expression of MPZL3 during tumor progression, we compared *MPZL3* transcript expression in ovarian and omental tumors to matched normal fallopian tube specimens from an independent tissue bank at the University of Pittsburgh. Similar to TCGA data, primary ovarian tumors were comprised of both high- and low-expressing populations, whereas the majority of omental tumors, which represents a major site of metastasis, displayed decreased MPZL3 expression (Fig. 1D), suggesting that a loss of MPZL3 is associated with tumor progression.

### EMT gene expression is significantly enriched following MPZL3 knockdown

**Figure 2:**
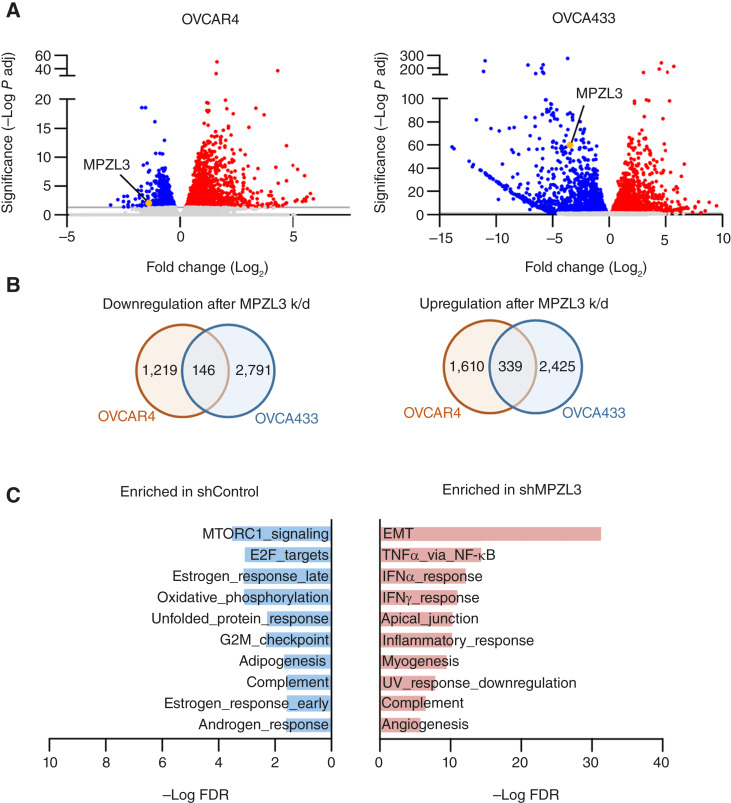
RNA-seq analysis following MPZL3 knockdown. **A,** Distribution of selected differentially expressed genes (DEG) in OVCAR4 and OVCA433 cells following MPZL3 knockdown (threshold: adjusted *P* value < 0.05, log_2_ fold change ≥1). **B,** Venn diagram of the number of significantly downregulated and upregulated DEGs in OVCA433 and OVCAR4 cells following MPZL3 knockdown. **C,** Enriched hallmark pathways commonly altered in OVCA433 and OVCAR4 cells following MPZL3 knockdown (MSigDB analysis of shared DEGs).

To better understand the role of MPZL3 in ovarian cancer, RNA-seq was carried out following MPZL3 knockdown in OVCAR4 and OVCA433 cells, two cell lines of high-grade serous origin, using two independent shRNAs each (Fig. 2A–C; Supplementary Fig. S2A). MSigDB analysis was carried out on differentially expressed genes (adjusted *P* value <0.05 and log_2_ fold change ≥1) that were significantly altered by both shRNAs and shared by OVCAR4 and OVCA433 cells following MPZL3 knockdown (Fig. 2B and C). Hallmark analysis revealed the strongest enrichment in EMT-related genes following MPZL3 knockdown, whereas cell cycle–associated gene sets including MTORC1, E2F, and G2M checkpoint were enriched in the control group (Fig. 2C; Supplementary Fig. S2D).

**Figure 3:**
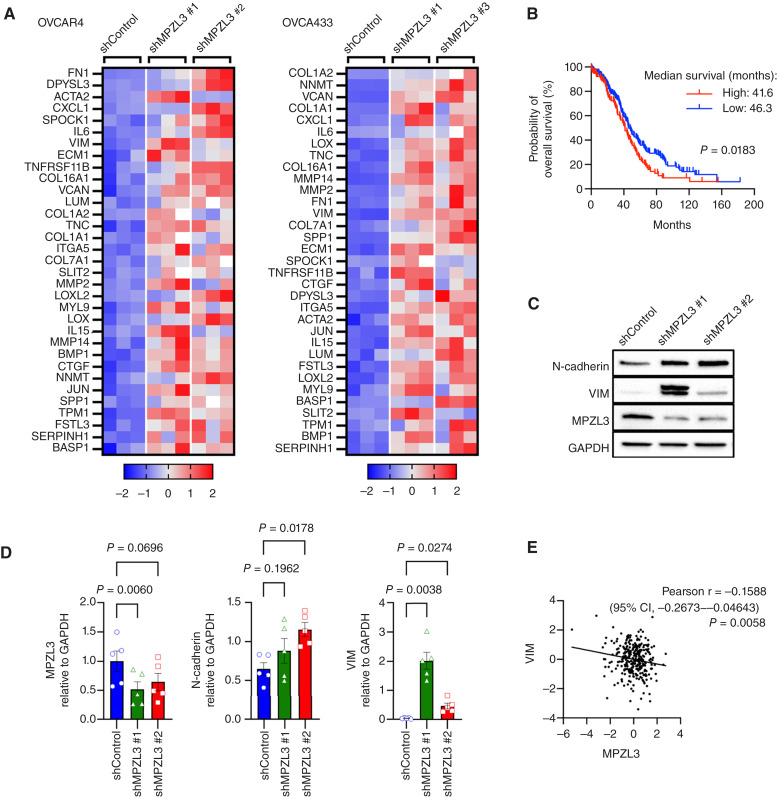
MPZL3 knockdown increases EMT gene expression signature which is associated with poor patient outcome. **A,** Heatmap of EMT signature gene transcripts increased in response to MPZL3 knockdown with two independent shRNAs in OVCAR4 and OVCA433 cells (RNA-seq, *z*-scores). **B,** Overall patient survival decreases with high expression of the MPZL3-dependent EMT gene signature (TCGA ovarian serous adenocarcinoma, log-rank Mantel–Cox test). **C,** Expression of VIM and N-cadherin protein levels are increased following MPZL3 knockdown in OVCAR4 cells. **D,** Densitometry quantification of VIM and N-cadherin expression from five independent immunoblot experiments [*n* = 5; one-way ANOVA *P* = 0.0115 (MPZL3), *P* = 0.0065 (N-cadherin), *P* = 0.0025 (VIM); Dunnett multiple comparison test *P* values shown]. **E,** VIM expression is negatively correlated to MPZL3 expression in TCGA ovarian serous adenocarcinoma specimens (mRNA *z*-scores; RNA-seq V2 RSEM). CI, confidence interval.

The role of CAMs in regulating EMT is well established (6, 35), but this has not been previously associated with MPZL3 loss. We found that the shared EMT signature gene set connected to MPZL3 knockdown in OVCAR4 and OVC433 cells (Fig. 3A) was associated with significantly worse overall patient outcomes (TCGA PanCancer Atlas; Fig. 3B). VIM and N-cadherin (CDH2) expression were further assessed at the protein levels in OVCAR4 cells following MPZL3 knockdown, which demonstrated a strong induction of VIM expression in response to decreased MPZL3 expression (Fig. 3C and D), similar to that observed at the transcript level (Fig. 3A; Supplementary Fig. S2E). Moreover, VIM expression was inversely correlated to MPZL3 expression in TCGA ovarian cancer specimens (Fig. 3E), indicating that decreased MPZL3 expression is associated with upregulation of the canonical EMT gene VIM in ovarian cancer. Morphologic changes consistent with EMT, including elongated, spindle-like cell shapes, were also observed following MPZL3 knockdown (Supplementary Fig. S2F), which was more evident in OVCAR4 than OVCA433 cells.

### Loss of MPZL3 reduces homotypic cell adhesion and increases cell invasiveness of OVCAR4 cells

**Figure 4:**
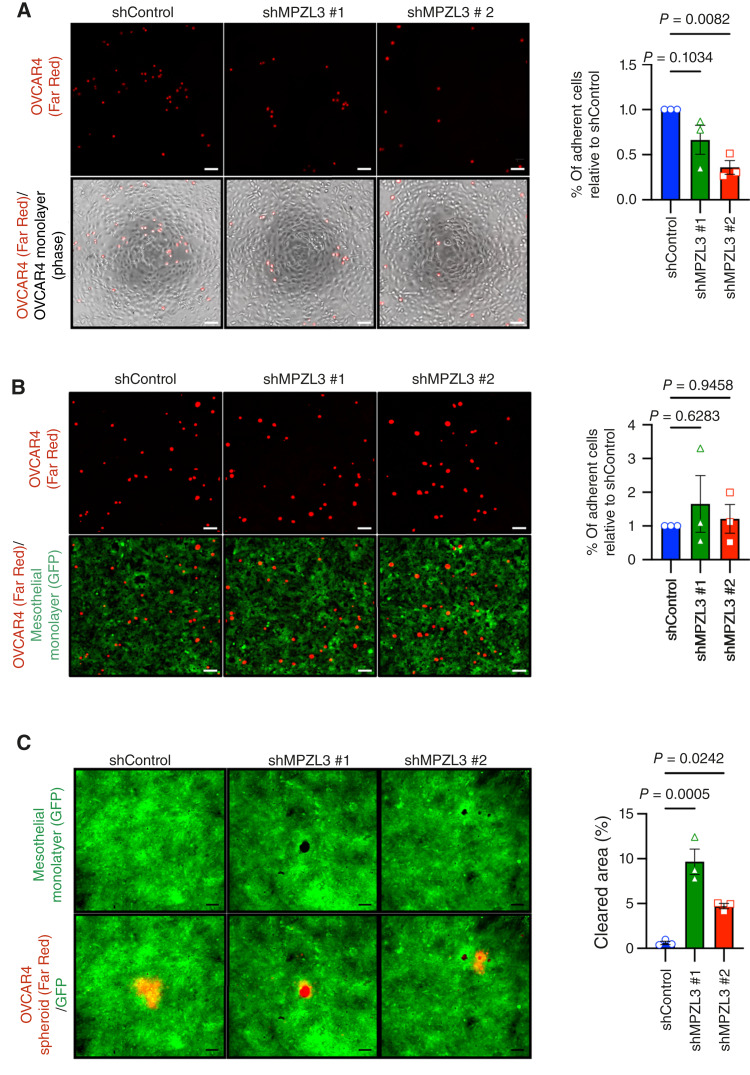
Loss of MPZL3 reduces homotypic cell adhesion and induces invasion of ovarian cancer cells. **A,** Homotypic adhesion was assessed by seeding parental OVCAR4 cells in a monolayer (phase-contrast image) and quantifying adherence of OVCAR4 MPZL3 knockdown cells labeled with Far Red fluorescent relative to the adherence of scr control cells (scale bar, 100 μm; *n* = 3; one-way ANOVA *P* = 0.0133; Dunnett multiple comparison test *P* values shown). **B,** Heterotypic cell adhesion was assessed as in **A**, except OVCAR4 scr control or MPZL3 knockdown cells labeled with Far Red fluorescent dye were placed on GFP-labeled mesothelial cells (*n* = 3; one-way ANOVA *P* = 0.7023; Dunnett multiple comparison test *P* values shown). **C,** GFP-labeled mesothelial cells were seeded in a monolayer, and mesothelial clearance by OVCAR4 scr control or MPZL3 knockdown spheroids labeled with Far Red fluorescent dye was quantified after 48 hours (scale bar, 100 μm; *n* = 3; one-way ANOVA *P* = 0.0008; Dunnett multiple comparison test *P* values shown).

To determine whether MPZL3 has CAM properties in ovarian cancer cells, we tested the effects of MPZL3 knockdown in OVCAR4 cells on homotypic cell adhesion by quantifying their attachment to a monolayer of parental OVCAR4 cells, and assessing their heterotypic adhesion to mesothelial cells. Mesothelial cells represent the first barrier of attachment and invasion ovarian cancer cells face during metastasis in the peritoneal cavity. Loss of MPZL3 decreased homotypic adhesion between ovarian cancer cells but did not affect heterotypic cell adhesion to mesothelial cells (Fig. 4A and B), suggesting that MPZL3 shares functional similarities to junctional adhesion molecules such as VSIG1. Finally, we conducted a mesothelial clearance assay (36), which demonstrated that MPZL3 knockdown promotes invasion and clearance of mesothelial cells (Fig. 4C). In summary, our findings demonstrate that loss of MPZL3 induces EMT, reduces homotypic cell adhesion, and promotes invasion of ovarian cancer cells.

### MPZL3 knockdown leads to cell-cycle arrest and senescence-like phenotype

**Figure 5:**
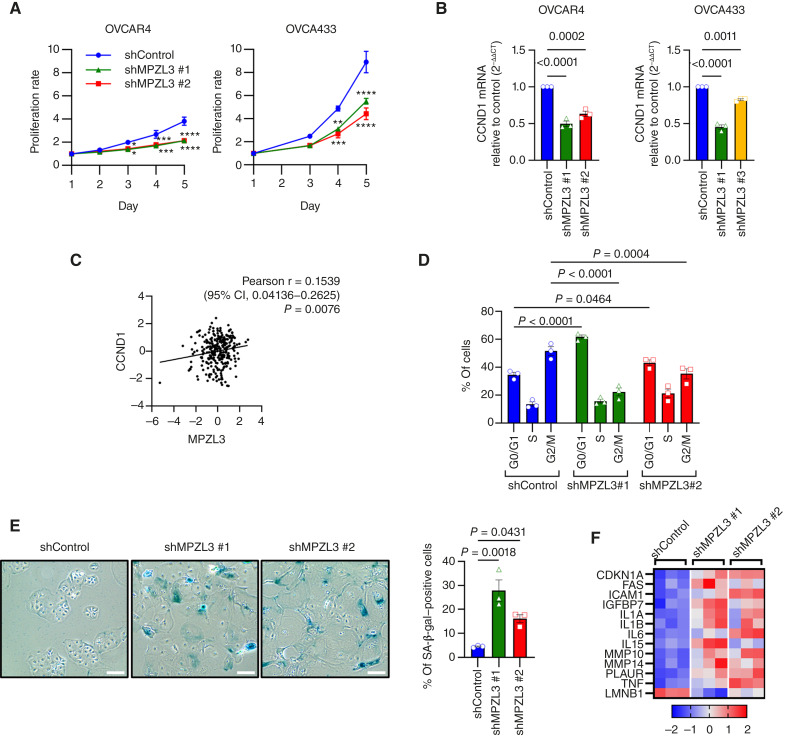
MPZL3 knockdown leads to cell-cycle arrest and senescence-like phenot. **A,** Cell growth assay following knockdown of MPZL3 in OVCAR4 and OVCA433 cells. Cell growth (FluoReporter Blue Fluorometric dsDNA Quantitation) was normalized to day 1 (*n* = 3; two-way ANOVA group factor variance *P* < 0.0001; Dunnett multiple comparison test *, *P* < 0.05; **, *P* < 0.01; ***, *P* < 0.001; ****, *P* < 0.0001). **B,** Semi-quantitative RT-PCR analysis of CCND1 transcript levels in OVCAR4 and OVCA433 cells following MPZL3 knockdown (*n* = 3; one-way ANOVA *P* < 0.0001; Dunnett multiple comparison test *P* values shown). **C,** CCND1 expression positively correlates with MPZL3 expression in TCGA ovarian serous adenocarcinoma specimens (mRNA *z*-scores; RNA-seq V2 RSEM). **D,** Cell-cycle analysis (propidium iodide flow cytometry) of OVCAR4 cells following MPZL3 knockdown at 9-hour timepoint from release (*n* = 3; two-way ANOVA group factor variance *P* < 0.0001; Dunnett multiple comparison test *P* values shown). **E,** Images of senescence-associated β-galactosidase (SA-β-gal) staining in OVCAR4 cells (scale bar, 200 μm). The corresponding quantification data are shown on the right (*n* = 3; one-way ANOVA *P* = 0.0029; Dunnett multiple comparison test *P* values shown). **F,** Heatmap of senescence-associated secretory phenotype markers and lamin B1 (LMNB1) following MPZL3 knockdown (OVCAR4 RNA-seq, *z*-scores). CCND1, cyclin D1; CI, confidence interval.

In addition to driving an EMT phenotype, MPZL3 knockdown led to a noticeable decrease in proliferation (Fig. 5A; Supplementary Fig. S3A). This correlated with a decrease in transcript expression associated with cell-cycle genes (Fig. 2C; Supplementary Fig. S2C and S3B), including cyclin D1 (Fig. 5B). Cyclin D1 and MPZL3 transcript expression were also significantly correlated in TCGA patient specimens (Fig. 5C), and knockdown of MPZL3 resulted in a slowing in G0/1 to S/G2/M transition (Fig. 5D; Supplementary Fig. S3C). These findings suggest that loss of MPZL3 in ovarian cancer cells leads to G1 cell-cycle arrest. We also observed that MPZL3 knockdown in OVCAR4 and OVCA433 cells led to a greater percentage of cells adopting a senescent-like phenotype, visualized using a β-galactosidase activity assay (Fig. 5E; Supplementary Fig. S3D), and this was accompanied by upregulation of common genes associated with the senescence-associated secretory phenotype (37–42), such as *IL6* and *IL1A.LMNB1* loss, another marker of senescence, was also observed (Fig. 5F). We note that a population of these cells is still proliferating, which may be due to heterogenous knockdown in the culture.

### Loss of MPZL3 decreases OVCAR4 sensitivity to cisplatin

**Figure 6:**
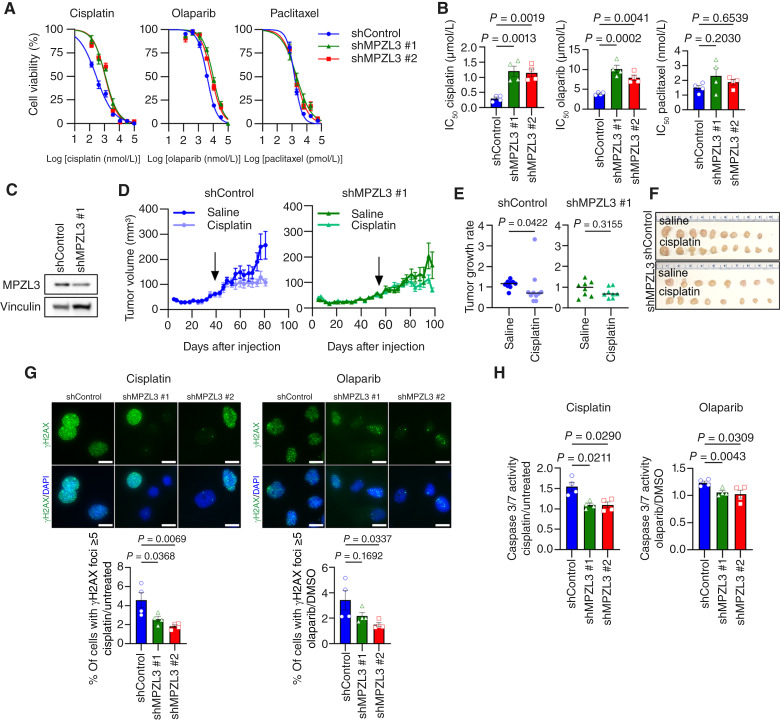
MPZL3 loss enhances cisplatin and olaparib resistance in OVCAR4 cells. **A,** Dose–response curves were derived from cell viability assays (FluoReporter dsDNA quantification) of OVCAR4 cells expressing scr control or MPZL3 shRNAs (*n* = 4, mean ± SEM). **B,** IC_50_ values derived from dose–response curves in **A** [*n* = 4; one-way ANOVA *P* = 0.0011 (cisplatin), *P* = 0.0003 (olaparib), and *P* = 0.2793 (paclitaxel); Dunnett multiple comparison test *P* values shown]. **C,** Western blot of MPZL3 knockdown OVCAR4 cells that were injected subcutaneously into Nod/SCID gamma mice. **D,** Subcutaneous tumor growth of OVCAR4 cells expressing scr control (left) or shMPZL3#1 (right) under vehicle (saline) or cisplatin treatment (two tumors per mouse, *n* = 4–5 mice per group). The start of cisplatin treatment is indicated with an arrow. **E,** Tumor growth rates under treatments for both scr control and shMPZL3#1 groups. Growth rates were calculated by dividing the final tumor volumes by the average tumor volumes from three measurements: 3 days before treatment, on the day treatment began, and 4 days after treatment began (median tumor volume shown, *n* = 8–10, Mann–Whitney test). **F,** Final tumor volume images of OVCAR4 subcutaneous tumors from both saline- and cisplatin-treated groups. All mice were euthanized after receiving the same doses of treatment. **G,** Immunofluorescence images and quantification of γH2AX signals induced by cisplatin (left) or olaparib (right) in OVCAR4 cells following MPZL3 knockdown [scale bar, 20 μm; *n* = 4; one-way ANOVA *P* = 0.0100 (cisplatin) and *P* = 0.0514 (olaparib); Dunnett multiple comparison test *P* values shown]. **H,** Caspase 3/7 activity induced by cisplatin (left) or olaparib (right) in OVCAR4 cells following MPZL3 knockdown [*n* = 4; repeated measures one-way ANOVA *P* = 0.0034 (cisplatin) and *P* = 0.0263 (olaparib); Dunnett multiple comparison test *P* values shown].

Given the observed slowing in proliferation, we hypothesized that loss of MPZL3 might increase drug resistance to chemotherapeutic agents. We determined the IC_50_ values of three standard-of-care compounds used to treat ovarian cancer in OVCAR4 cells, which are *TP53*-mutant but do not harbor *BRCA1* or *BRCA2* mutations, and observed that MPZL3 knockdown promoted resistance to cisplatin and to a lesser extent olaparib, whereas no significant difference was observed with paclitaxel treatment (Fig. 6A and B). However, this effect seemed to be cell type–specific as MPZL3 knockdown did not affect OVCA433 sensitivity to cisplatin or olaparib (Supplementary Fig. S4). These data suggest that MPZL3 depletion may decrease drug sensitivity in some cell lines in response to treatments that induce DNA damage (43, 44). To determine whether the increased cisplatin resistance observed in MPZL3-depleted OVCAR4 cells is recapitulated *in vivo*, we subcutaneously injected OVCAR4 cells expressing scr control or MPZL3 shRNA#1 into Nod/SCID gamma mice (Fig. 6C–F). Once the average tumor volume in each group reached approximately 50 mm^3^, mice were treated with either vehicle (saline) or cisplatin (Supplementary Fig. S5A). As expected from cell culture assays (Fig. 5), MPZL3 knockdown tumors grew slower than scr controls (Fig. 6D and E). Although the effects *in vivo* are subtle, comparing tumor growth rates in response to cisplatin treatment, the scr control group significantly decreased their growth rate in response to cisplatin treatment, whereas a significant change was not observed following MPZL3 knockdown (Fig. 6E).

To further investigate how MPZL3 knockdown causes resistance to cisplatin and olaparib, γH2AX foci were assessed by immunofluorescence to monitor DNA damage response. Both cisplatin and olaparib treatments led to a higher percentage of cells with multiple γH2AX foci in scr control cells compared with MPZL3 knockdown cells, indicating that MPZL3-depleted cells accumulate less DNA damage in response to cisplatin or olaparib stimulation (Fig. 6G). In addition, cisplatin and olaparib treatments induced higher levels of caspase 3/7 activity in control cells compared with MPZL3 knockdown cells (Fig. 6H). Western blot analysis on frozen tumor tissues obtained from the above *in vivo* experiment also showed that MPZL3 knockdown tumors grown under saline control treatment conditions exhibited reduced levels of γH2AX compared with controls, suggesting a lower baseline level of DNA damage in the absence of MPZL3 (Supplementary Fig. S5B and S5C). At endpoint, γH2AX levels were no longer readily detectable in the little remaining tumor tissue following cisplatin treatment. The above data demonstrate that MPZL3-deficient cells may be less susceptible to DNA damage and apoptosis and that this could contribute to decreased cisplatin chemosensitivity in some ovarian cancer cell lines.

## Discussion

In this study, we investigated the role of MPZL3 in ovarian cancer. CNA analysis revealed that the *MPZL3* locus, 11q23.3, is frequently altered across multiple cancer types (Fig. 1). Whereas this chromosomal region has been reported to be lost in many cancers (18–21), suggesting a tumor-suppressor function for MPZL3, previously published bioinformatics data have suggested that MPZL3 is a prognostic marker in breast cancer (22). We found a similar percentage of chromosome gains and losses at the *MPZL3* locus in the TCGA ovarian adenocarcinoma dataset (Supplementary Fig. S1A), leading us to question how MPZL3 influences ovarian cancer development and progression. It remains to be determined how this interpatient heterogeneity related to *MPZL3* locus gain and loss reflects on MPZL3 expression levels during different stages of ovarian cancer initiation, progression, and metastasis. Our limited in-house patient specimen data suggest that MPZL3 expression is highly heterogeneous in ovarian tumors, whereas omental tumors display primarily decreased expression. This suggests that MPZL3 loss may be a feature of tumor progression and metastatic disease (Fig. 1D).

From our unbiased RNA-seq analysis performed on two ovarian cancer cell lines following MPZL3 knockdown, we observed that EMT gene sets were the most significantly enriched in the knockdown group, and that cell proliferation and cell-cycle gene sets were predominantly expressed in the control group (Fig. 2). MPZL3 depletion reduced homotypic cell adhesion and enhanced mesothelial clearance and invasion in OVCAR4 cells (Fig. 4) while decreasing proliferation (Fig. 5). Further work is needed to establish whether MPZL3 acts directly as a CAM, as it contains an IgV domain, or if it alters adhesion indirectly in epithelial cells, and how MPZL3 loss drives an EMT phenotype in cancer cells. Multiple CAMs have been shown to drive cancer progression by regulating cell–cell and cell–matrix interactions or acting as receptors for downstream signaling, ultimately influencing tumor growth and metastasis (35, 45, 46). Both the loss and gain of specific CAMs are important features of EMT and metastasis. Our data suggests that loss of MPZL3 is reminiscent of the changes in E-cadherin observed during EMT and cancer metastasis. Although the precise signaling pathways downstream of MPZL3 have not been fully characterized, previous studies have implicated MPZL3 as necessary for metabolic changes, epidermal barrier formation, and hair follicle development (8–15). In our study, MPZL3 knockdown led to increased VIM and N-cadherin levels, hallmarks of EMT, suggesting that MPZL3 may contribute to maintaining epithelial phenotypes and suppressing mesenchymal features. Given its potential role as a CAM, it is plausible that MPZL3 contributes to the stabilization of cell–cell junctions and that MPZL3 loss may thus indirectly modulate signaling pathways associated with EMT, such as alterations in FAK, PI3K/AKT, or MAPK pathways. Alternatively, MPZL3 might influence cell adhesion by directly interacting with other CAMs, such as E-cadherin and N-cadherin. A closely related homolog of MPZL3, MPZL2 (EVA1) localizes with E-cadherin and ZO-1 at cell–cell junctions in breast cancer epithelial cells. Similar to our observations, MPZL2 knockdown results in decreased E-cadherin and increased N-cadherin levels and an EMT phenotype (47). To better understand and draw further conclusions on the role of MPZL3 as a *bona fide* CAM, future studies are needed to elucidate novel binding partners of MPZL3 in normal and cancer epithelial cells with focus on the N-terminal extracellular IgV and C-terminal cytoplasmic domains. In this study, we attempted to express MPZL3 with a C-terminal V5 tag but were unable to detect MPZL3 protein using an MPZL3-specific antibody. We suspect that the tag interfered with proper MPZL3 folding, expression, and/or function.

Our findings demonstrating that MPZL3 loss drives both EMT and decreased proliferation are not surprising because a negative association between EMT and cell-cycle progression has been reported (48). We attempted to generate complete MPZL3 knockout cells using CRISPR/Cas9 in OV90 cells but were unable to derive knockout clones. It is possible that cells have a metastatic advantage with low MPZL3 expression but that complete loss is detrimental due to a loss of proliferation. Thus, the levels of MPZL3 expression may be indicative of changes between proliferative and metastatic cell populations within tumors cells. Moreover, the connection between senescence, EMT, and drug resistance has also previously been established (42, 49–52). Our data revealed that MPZL3 loss induces some cells to enter a senescent-like state and increases expression of senescence-associated secretory phenotype genes (Fig. 5). In addition, we found increased resistance to cisplatin and olaparib in OVCAR4 cells following MPZL3 loss, two compounds that elicit DNA damage or target DNA repair (Fig. 6). Resistance to these compounds in MPZL3-depleted cells seems to be due to their decreased proliferation, resulting in reduced DNA damage accumulation and thus decreased apoptosis. This was somewhat surprising as senescence is generally associated with increased DNA damage, yet we conversely observed decreased DNA damage in response to MPZL3 knockdown in OVCAR4 tumor tissues and in cell culture in response to these compounds. Given that CAMs are implicated in both DNA damage response and drug resistance (53, 54), how MPZL3 loss may enhance DNA repair capacity requires further investigation. Although these findings are intriguing, it should be pointed out that chemoresistance was not observed in OVCA433 cells, which may be due to differences in genetic background and inherent drug resistance mechanisms that may be independent of MPZL3 dependency. Moreover, the effects of MPZL3 loss on OVCAR4 chemosensitivity were less striking *in vivo* (Fig. 6), and this is likely attributable to additional factors, including interactions with the tumor microenvironment, that are absent *in vitro*.

Further studies are also necessary to test the effects of MPZL3 loss in an immune-competent *in vivo* model, given the enrichment of immune-associated pathways observed in our RNA-seq analysis (Fig. 2C). Moreover, MPZL3 contains an Ig-like domain, which may be involved in mediating interactions between cancer cells and immune cells (55, 56). However, the specific roles and mechanisms of MPZL3 in the TME, particularly in tumor immune infiltration in cancer, have not yet been studied. We analyzed the correlation between MPZL3 expression and immune infiltration levels using TIMER2.0 (57–59) and found that MPZL3 is positively correlated with CD8^+^ T cells, cancer-associated fibroblasts, and neutrophils and negatively correlated with B cells in ovarian cancer (Supplementary Fig. S6). Thus, it is possible that a loss of MPZL3 could also influence the recruitment of tumor-associated immune cells.

We and others have previously implicated MPZL3 in the regulation of adipogenesis *in vivo* (8, 12). Further studies are needed to determine whether loss of MPZL3 similarly affects lipid metabolism in ovarian cancer cells. Notably, our RNA-seq data show that oxidative phosphorylation and adipogenesis gene set were enriched in control cells. We previously found that MPZL3 antisense knockdown can protect mice from diet-induced obesity and decrease adiposity and circulating lipids (8). Interestingly, one of the target genes of MPZL3 loss, VIM, has been implicated in lipolysis, lipid uptake, and lipid droplet transport (60–62). Although somewhat contradictory, *VIM*-/- mice seem to display a similar metabolic phenotype compared with MPZL3-/- mice (63, 64). Nevertheless, whether changes in VIM also drive altered lipid metabolism in ovarian cancer cells is an intriguing hypothesis, particularly because lipid metabolism is a phenotype of tumor cells metastasizing to the omentum.

These data demonstrate for the first time that loss of MPZL3 results in EMT-associated gene expression, decreases proliferation, and can selectively affect drug sensitivity in ovarian cancer. Thus, our data suggest that decreased MPZL3 expression may be a phenotype of ovarian cancer tumor progression and metastasis.

## Supplementary Material

Supplementary Figure S1 Supplementary Figure S1. Percentage of MPZL3 CNA and CNA based overall survival in TCGA ovarian adenocarcinoma dataset.

Supplementary Figure S2 Supplementary Figure S2. MPZL3 knock-down affects transcription of EMT and cell cycle-related genes in ovarian cancer cells.

Supplementary Figure S3 Supplementary Figure S3. MPZL3 knock-down decreases proliferation and cell cycle progression.

Supplementary Figure S4 Supplementary Figure S4. Dose-response curves of cisplatin and olaparib in OVCA433 cells following MPZL3 knockdown.

Supplementary Figure S5 Supplementary Figure S5. γH2AX expression in tumor tissues from in vivo experiments.

Supplementary Figure S6 Supplementary Figure S6. Association between MPZL3 expression and immune cell infiltration.
