# Supplementary material for: Loss of Predicted Cell Adhesion Molecule MPZL3 Promotes EMT in Ovarian Cancer
Source: Cancer Res Commun. 2025 Jul 21;5(7):1180–93. doi: 10.1158/2767-9764.CRC-24-0591 (PMC12277487; doi:10.1158/2767-9764.CRC-24-0591)
Supplement: Supplementary Figure S1 — Percentage of MPZL3 CNA and CNA based overall survival in TCGA ovarian adenocarcinoma dataset. [file crc-24-0591_supplementary_figure_s1_suppsf1.pdf]

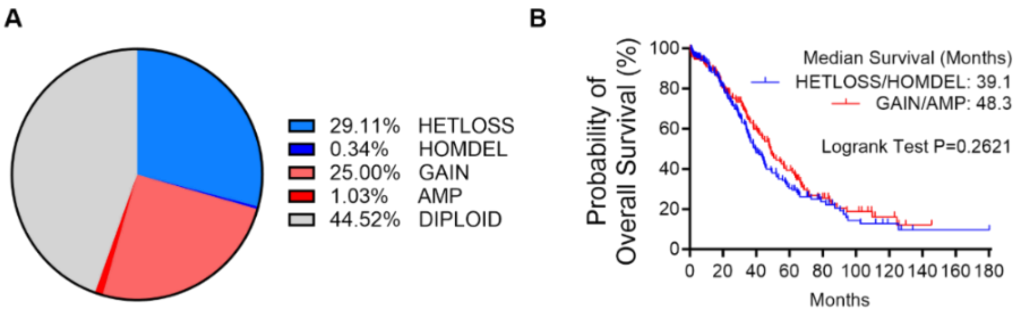

Supplementary Figure S1. Percentage of *MPZL3* CNA and CNA based overall survival in TCGA ovarian adenocarcinoma dataset.

A. Percentage of various MPZL3 CNA in ovarian adenocarcinoma samples (TCGA, PanCancer Atlas).
B. Overall patient survival in relation to *MPZL3* copy number. Loss of MPZL3 is negatively associated with patient survival (Logrank Mantel-Cox test).
