# Supplementary material for: Loss of Predicted Cell Adhesion Molecule MPZL3 Promotes EMT in Ovarian Cancer
Source: Cancer Res Commun. 2025 Jul 21;5(7):1180–93. doi: 10.1158/2767-9764.CRC-24-0591 (PMC12277487; doi:10.1158/2767-9764.CRC-24-0591)
Supplement: Supplementary Figure S2 — MPZL3 knock-down affects transcription of EMT and cell cycle-related genes in ovarian cancer cells. [file crc-24-0591_supplementary_figure_s2_suppsf2.pdf]

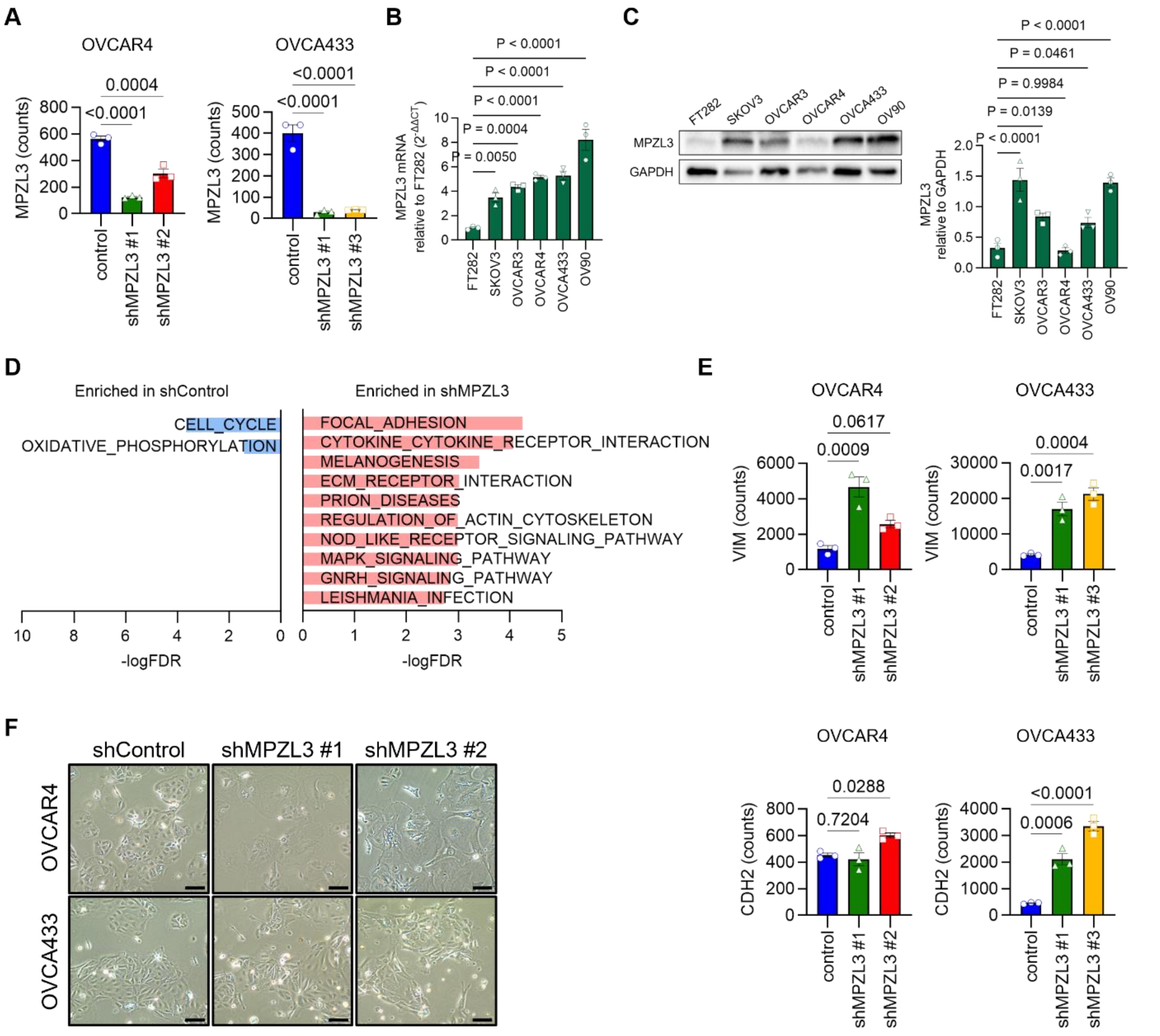

Supplementary Figure S2. MPZL3 knock-down affects transcription of EMT and cell cycle-related genes in ovarian cancer cells.

A. MPZL3 expression following shRNA mediated knock-down in OVCAR4 and OVCA433 (counts from RNA-seq analysis) (n=3; one-way ANOVA P<0.0001; Dunnett's multiple comparisons test P values shown).
B. *MPZL3* mRNA expression in ovarian cancer cell lines (n=3; one-way ANOVA P<0.0001; Dunnett's multiple comparisons test P values shown)
C. Western blot analysis of MPZL3 protein levels across multiple ovarian cancer cell lines. Densitometric quantification of MPZL3 expression based on three independent biological replicates (n = 3; one-way ANOVA P<0.0001; Dunnett's multiple comparisons test P values shown).
D. Enriched KEGG pathways commonly altered in OVCA433 and OVCAR4 cells following MPZL3 knockdown (MSigDB analysis of shared DEGs).
E. Vimentin (VIM) and N-cadherin (CDH2) expression in OVCAR4 and OVCA433 cells following MPZL3 knock-down (RNA-seq; n=3; one-way ANOVA P=0.0015 (VIM, OVCAR4), P=0.0005 (VIM, OVCA433), P=0.0151 (CDH2, OVCAR4), P<0.0001 (CDH2, OVCA433); Dunnett's multiple comparisons test P values shown).
F. Phase-contrast images showing the morphology of OVCAR4 and OVCA433 cells following MPZL3 knock-down (scale bar: 200 μm).
