# Supplementary material for: Loss of Predicted Cell Adhesion Molecule MPZL3 Promotes EMT in Ovarian Cancer
Source: Cancer Res Commun. 2025 Jul 21;5(7):1180–93. doi: 10.1158/2767-9764.CRC-24-0591 (PMC12277487; doi:10.1158/2767-9764.CRC-24-0591)
Supplement: Supplementary Figure S3 — MPZL3 knock-down decreases proliferation and cell cycle progression. [file crc-24-0591_supplementary_figure_s3_suppsf3.pdf]

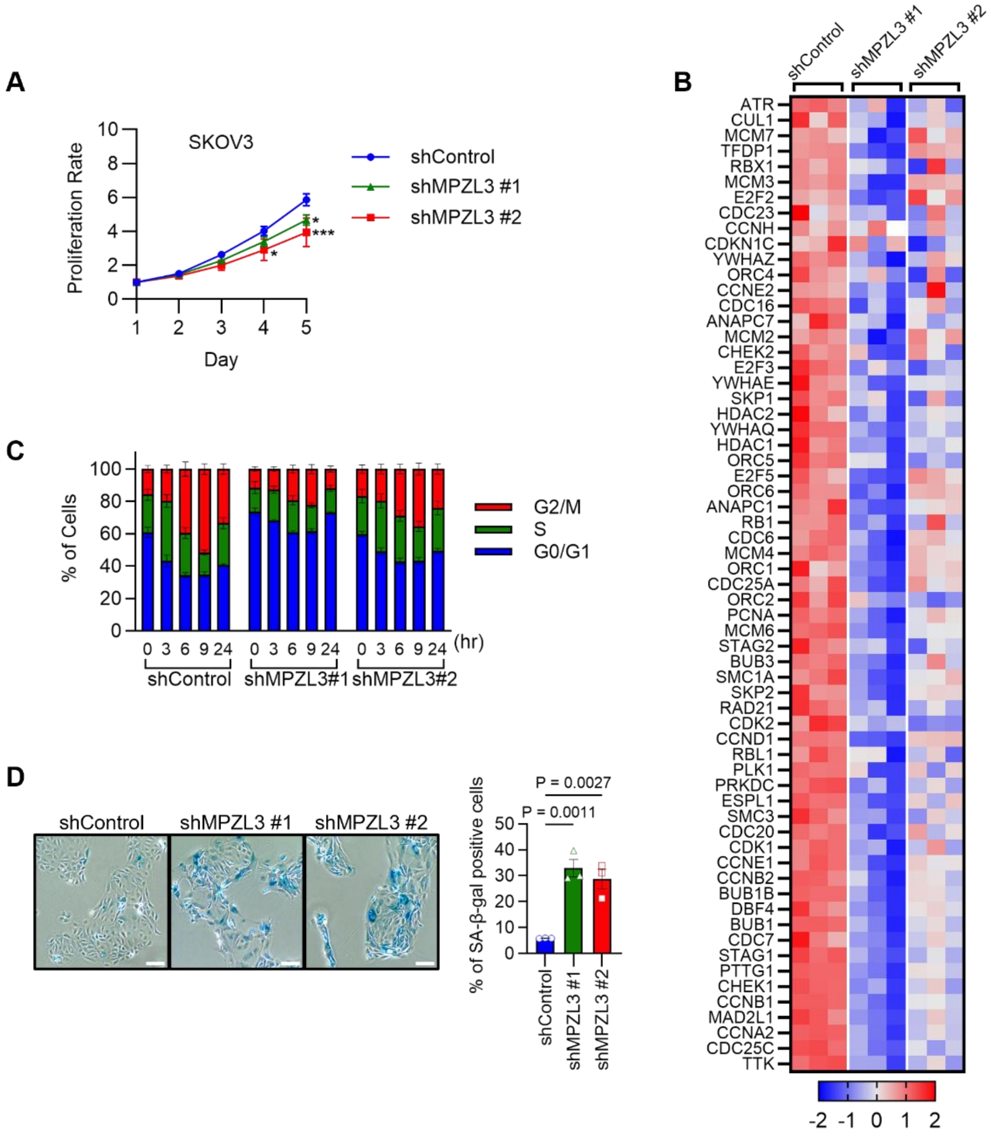

Supplementary Figure S3. MPZL3 knock-down decreases proliferation and cell cycle progression

A. Cell growth assay results for SKOV3 cells following MPZL3 knock-down. Results were normalized to day 1 to obtain the normalized ratio (N=3; two-way ANOVA group factor variance P=0.2240; Dunnett's multiple comparisons test *: P<0.05, **: P<0.01, ***: P<0.001, ****: P<0.0001).
B. Heatmap of core enrichment genes from the cell cycle gene set following MPZL3 knockdown (KEGG, OVCAR4 RNA-seq, z-scores).
C. PI Flow cytometry analysis in OVCAR4 cells with MPZL3 knockdown. Cells were synchronized at the G1/S phase, released and harvested at 0, 3, 6, 9, and 24 hrs (n=3).
D. Images of senescence-associated (SA) β-galactosidase staining in OVCA433 cells (scale bar: 200 μm). The corresponding quantification data are shown in the right panel. (N=3; one-way ANOVA P=0.0013; Dunnett's multiple comparisons test P values shown).
