# Supplementary material for: Loss of Predicted Cell Adhesion Molecule MPZL3 Promotes EMT in Ovarian Cancer
Source: Cancer Res Commun. 2025 Jul 21;5(7):1180–93. doi: 10.1158/2767-9764.CRC-24-0591 (PMC12277487; doi:10.1158/2767-9764.CRC-24-0591)
Supplement: Supplementary Figure S4 — Dose-response curves of cisplatin and olaparib in OVCA433 cells following MPZL3 knockdown. [file crc-24-0591_supplementary_figure_s4_suppsf4.pdf]

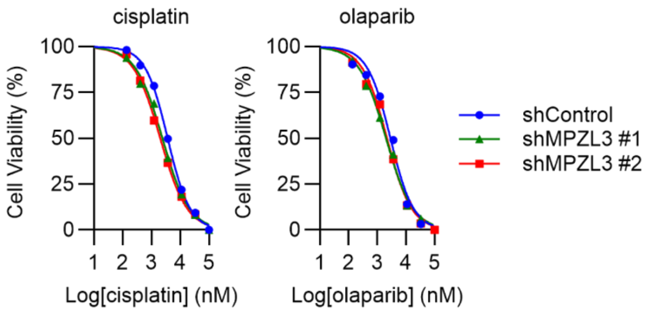

Supplementary Figure S4. Dose-response curves of cisplatin and olaparib in OVCA433 cells following MPZL3 knockdown.

Dose-response curves were generated from cell viability assays (FluoReporter dsDNA quantification) in OVCA433 cells expressing scramble control or MPZL3 shRNAs (n = 4; mean ± SEM).
