# Supplementary material for: Loss of Predicted Cell Adhesion Molecule MPZL3 Promotes EMT in Ovarian Cancer
Source: Cancer Res Commun. 2025 Jul 21;5(7):1180–93. doi: 10.1158/2767-9764.CRC-24-0591 (PMC12277487; doi:10.1158/2767-9764.CRC-24-0591)
Supplement: Supplementary Figure S5 — γH2AX expression in tumor tissues from in vivo experiments. [file crc-24-0591_supplementary_figure_s5_suppsf5.pdf]

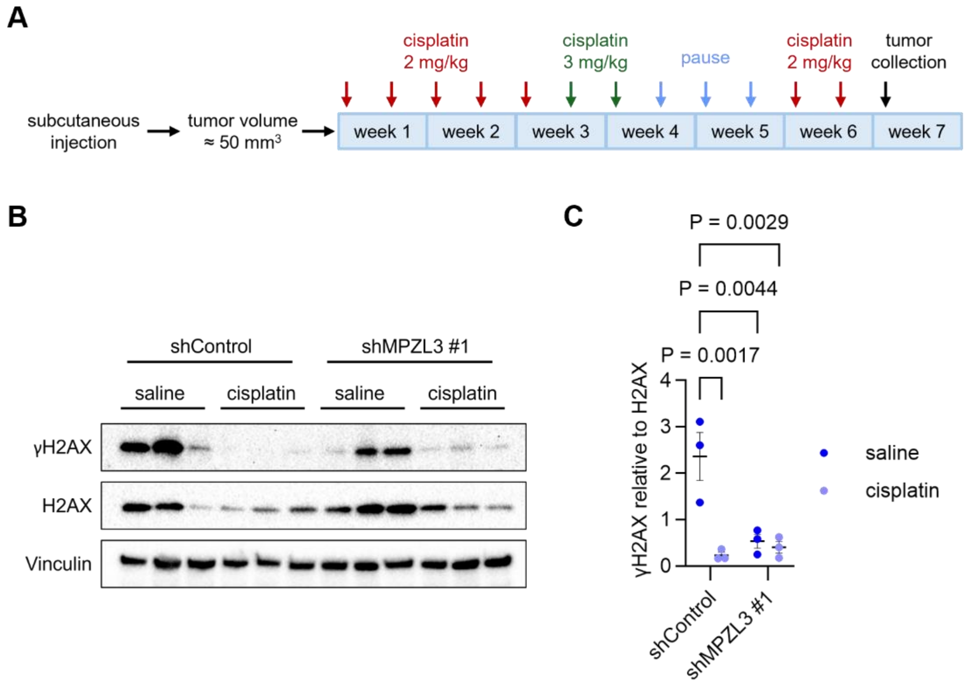

Supplementary Figure S5. γH2AX expression in tumor tissues from *in vivo* experiments.

A. Cisplatin treatment dosing schedule and experimental timeline.
B. Western blot analysis of γH2AX levels in tumor tissues from shControl and shMPZL3 #1 groups, treated with either saline or cisplatin. Three tumors were analyzed per treatment group.
C. Densitometric quantification of γH2AX expression from panel B (n = 3; two-way ANOVA group factor variance P=0.0072; Dunnett's multiple comparisons test P values shown).
