# Supplementary material for: Loss of Predicted Cell Adhesion Molecule MPZL3 Promotes EMT in Ovarian Cancer
Source: Cancer Res Commun. 2025 Jul 21;5(7):1180–93. doi: 10.1158/2767-9764.CRC-24-0591 (PMC12277487; doi:10.1158/2767-9764.CRC-24-0591)
Supplement: Supplementary Figure S6 — Association between MPZL3 expression and immune cell infiltration. [file crc-24-0591_supplementary_figure_s6_suppsf6.pdf]

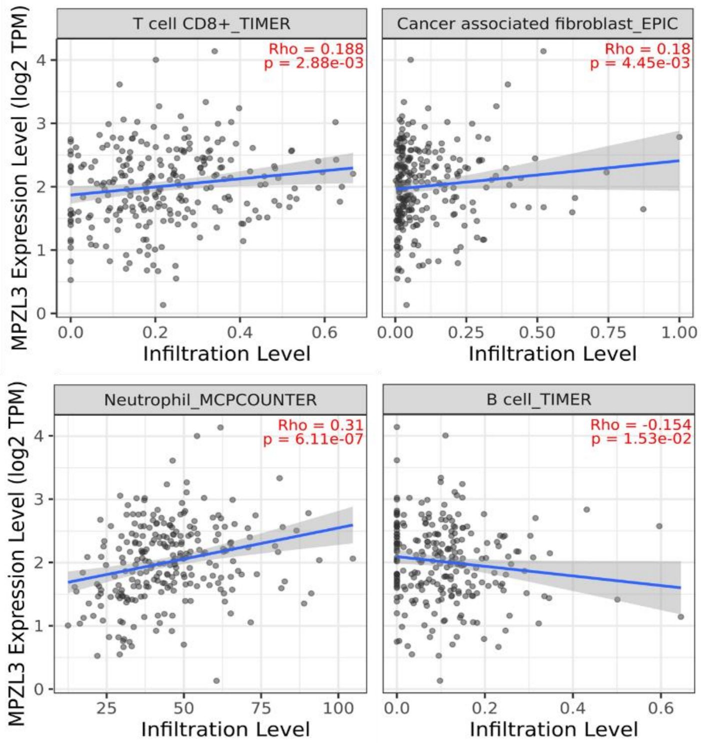

Supplementary Figure S6. Association between MPZL3 expression and immune cell infiltration.

Correlation between MPZL3 expression and infiltration of CD8+ T cells, cancer-associated fibroblasts, neutrophils, and B cells in TCGA serous ovarian cancer specimens, analyzed using TIMER, EPIC, MCP-COUNTER, and TIMER algorithms, respectively.
